# A Comparative Study of Morphometric Analysis of Nucleolar Organizer Regions in Oral Leukoplakia and Oral Squamous Cell Carcinoma and Significance of AgNOR as a Diagnostic Tool

**DOI:** 10.7759/cureus.44228

**Published:** 2023-08-27

**Authors:** Arun Singh, Shalini Singh, Virendra Soni, Dhirendra k Srivastava

**Affiliations:** 1 Dentistry, Prasad Institute of Medical Science and Hospital, Lucknow, IND; 2 Preventive Medicine, Baba Raghav Das (BRD) Medical College, Gorakhpur, IND

**Keywords:** silver nitrate, profile area per nucleus, nuclear profile area, microscopic fields, morphometric analysis, nucleolar organizer region, dysplasia, silver colloid, oral squamous cell carcinoma, oral leukoplakia

## Abstract

Background

Silver stainable nucleolar organizer regions (AgNORs) have proven to exhibit utmost importance due to their higher occurrence in the nucleus especially in malignant cells than in normal. Thus, they assist in the examination of nucleolar structures and variations in nucleolar activity.

Aim

Quantitative and qualitative analysis in relation to the number and area of AgNOR in tissue sections of the normal oral mucosa (NOM), oral leukoplakia (OL), and oral squamous cell carcinoma (OSCC) was the main aim of the study.

Materials & method

A total of 50 cases comprising 20 OL with dysplasia, 20 OSCC cases, and 10 samples of normal oral mucosa were taken. Silver nitrate (Sol A) & gelatin (Sol B) solutions were freshly prepared for staining the lesional slides.

Results

The mean value of nuclear profile area (A Nuc) was comparatively higher in oral leukoplakia i.e. 41.97 and in oral squamous cell carcinoma i.e. 62.36 in comparison to the control group where it was 36.19. The mean value of a single AgNOR profile area per nucleus (A NOR) was found to be comparatively lower in both study groups i.e. oral leukoplakia (2.76) and oral squamous cell carcinoma (1.61) in comparison to the control group (3.45) . The mean value of total AgNOR profile area per nucleus (TA NOR) and the number of profiles of AgNORs per nucleus (n NOR) were found higher in both study groups (oral leukoplakia and oral squamous cell carcinoma) as compared to normal oral mucosa of the control group.

However, the findings of all four parameters of morphometric analysis were found to be significantly associated with disorder of oral mucosa i.e. cases of oral leukoplakia and oral squamous cell carcinoma (P value <0.01).

Conclusion

It can thus be suggested that the mean AgNOR count displayed a higher value in OSCC. Hence, the number of AgNORs in nuclei increases as epithelial cells undergo malignant transformation which is designated that mean AgNOR count may contribute to establishing the prognosis of a lesion.

## Introduction

Squamous cell carcinoma (SCC) is one of the most common malignant neoplasms affecting the oral cavity, representing around 90% of all oral malignancies. Oral squamous cell carcinoma (OSCC) is an important cause of morbidity and mortality worldwide epitomizing a major health concern constituting up to 40% of other cancers in India [1]. Oral leukoplakia (OL) is the most common potentially malignant disorder with a propensity to transform into oral cancer [2]. Up to 80% of leukoplakias are benign with no evidence of dysplasia and no predisposition to malignancy but biopsy is indicated to define the remaining 10-20% that are either dysplastic or already invasive carcinomas. The potential for malignancy appears higher in certain sites (floor of mouth/ventral side of the tongue, lower lip, commissures) where the lesion is associated with Candida species, or where the lesion is verrucous or mixed with red lesions (erythroleukoplakia or speckled leukoplakia) [3].

OSCC is an epithelial neoplasia characterized by a localized clonal expansion of altered stem cells close to the basement membrane that grows laterally and upward, displacing the normal epithelium [4]. In Southeast Asia and Central Asia where the habit of tobacco or betel-tobacco chewing prevails, buccal involvement has been reported to be as high as 80% of the oral cancers [5].

Silver stainable nucleolar organizer regions (AgNORs) have acquired importance owing to their frequency within the nucleus being higher in malignant cells than in normal ones. Thus, they aid in the examination of nucleolar structures and variations in nucleolar activity. The chromosomal nucleolar organizer regions (NORs) are loops of ribosomal DNA (rDNA), which occur on the nuclei of cells possessing the genes for the synthesis of ribosomal RNA (rRNA). In humans, they are located on the short arm of the five acrocentric chromosomes 13, 14, 15, 21, and 22 [6].

The expression of AgNOR designates the silver stainable nucleolar organizer region proteins. The precise biochemistry of AgNOR has not been fully illustrated however, they have been categorized as B23, C23, AgNOR protein, and RNA polymerase [6]. The nature of nucleolar organizer regions-associated proteins (NORAPS) is relatively indeterminate but they possibly act as regulators of rDNA transcription or maintain the extended configuration of DNA. Nucleolar organizer regions (NOR) staining thus denotes actively transcribing NORs (rDNA) and the frequency of NORs per nucleus may prove to be a useful replicatory marker [7]. Computerized image analysis of a number of AgNORs, AgNOR area, size, and shape are the better-agreed methods to obtain an accurate and reproducible marker of the proliferative potential and cell stability of the tissue analyzed [8]. AgNOR expression is not a predictor of developing cell quantity or growth fraction. It reflects the cell cycle rate and is related to tumor doubling time. The morphometric technique is simple, fast, and reliable when computer-assisted area measurement is used. This might be an adjunct to the diagnostic possibilities for determining tumor behavior.

The current study aimed to compare the morphometric analysis of nucleolar organizer regions in oral leukoplakia and oral squamous cell carcinoma with normal oral mucosa. The clinical significance of the silver colloid technique, morphometric analysis, and highlights of AgNOR importance has also been studied here to serve as a diagnostic tool in OL and OSCC.

## Materials and methods

This study was undertaken in the Department of Oral Pathology & Microbiology at Sardar Patel Post Graduate Institute of Dental and Medical Sciences, Lucknow (SPPGIDMS). It was a case-control study. The study group comprised the diagnosed cases of OL (with dysplasia) and OSCC. Cases were randomly selected from the outpatient department of oral medicine and radiology, SPPGIDMS. The patients were clinically examined by the senior faculty members and clinical data pertaining to each case was collected. Patients with non-irradiated OSCC and histologically confirmed cases of OL were included in the study. A total of 20 cases of OL, and 20 cases of OSCC were taken as the study group, and 10 samples of normal oral mucosa (NOM) were taken as the control group. The study was approved by the ethical committee of the institute. The patients were clinically examined and written consent was obtained from them. Biopsy was undertaken in the most representative lesional sites.

The statistical analysis was done using SPSS (SPSS Inc. Released 2007. SPSS for Windows, Version 15.0. Chicago, SPSS Inc). The values were represented in number, percentage (%), and mean and standard deviation (SD) were calculated. The analysis of variance (ANOVA) test was used to compare the differences among the groups. To test the significance of two means the student "t " test was used.

Silver colloid technique solution was composed of two types of solutions - solution A and solution B. Solution A contained 25 gm of silver nitrate in 100 ml of distilled water and solution B contained one gm of gelatin and one ml of formic acid in 100 ml of distilled water. Two part of solution A and one part of solution B was taken as a working solution.

All the solutions were freshly prepared before use. The software for image analysis was done using Image Pro Express 6.0 Media Cybernetics, USA.

Silver colloid technique staining

AgNOR staining was done according to the modified procedure by Smith et al. [9]. Five-micron-thick sections of the paraffin blocks were deparaffinized, cleared in xylene, and hydrated through descending grades of alcohol till distilled water. Solutions A and B were prepared and the final working solution was obtained. This was immediately poured over the tissue sections and left for 40 minutes at room temperature in a dark place. The silver colloid was then washed off with distilled water and the sections were dehydrated in ascending grades of alcohol and then kept in a solution of absolute alcohol and xylene (in a 1:1 ratio) for five minutes. These sections were used to measure the nuclear area, and AgNOR area and count the AgNOR dots. AgNORs - black dots (intranuclear); other parts of nuclei - yellow-brown.

Morphometric analysis

Nuclear and AgNOR Measurements

The images were captured and the actual measurements were done using the Windows-based image analyzer software. Accurate calibration for magnification was done using a stage micrometer prior to measurements. The final image captured on the monitor had a magnification of X100. Microscopic fields were selected randomly, commencing with the first representative field on the left-hand side and then moving the stage to the next field and continuing the selection to include five fields from each section. Stage readings were noted for reassessment. From each field, five of the largest nuclei with clear outlines were traced with the pointer using the mouse. The numbers of silver-stained, black, or brownish-black dots, were measured in each nucleus. AgNOR dots seen in a clump were counted as single AgNOR dot. The nuclear area was automatically calculated by the software.

Nuclear Profile Area (A Nuc)

The nuclear perimeter was traced and the nuclear area was calculated (in square microns).

Single AgNOR Profile Area Per Nucleus (A NOR)

The AgNOR perimeter was traced and the software calculated the single AgNOR area (square microns). Here area of the total number of AgNORs per nucleus was calculated using morphometric analysis and the area of all AgNORs of 25 nuclei of five different fields was added and then divided by the total number of AgNORs of all 25 nuclei.

Total AgNOR Profile Area Per Nucleus (TA NOR)

The total AgNOR area per nucleus is calculated by using morphometric analysis and the area of all AgNOR of all 25 nuclei was obtained similarly.

Number of Profile of AgNORs per Nucleus (n NOR)

The number of profiles of AgNOR per nucleus (n NOR) was observed.

## Results

**Figure 1:**
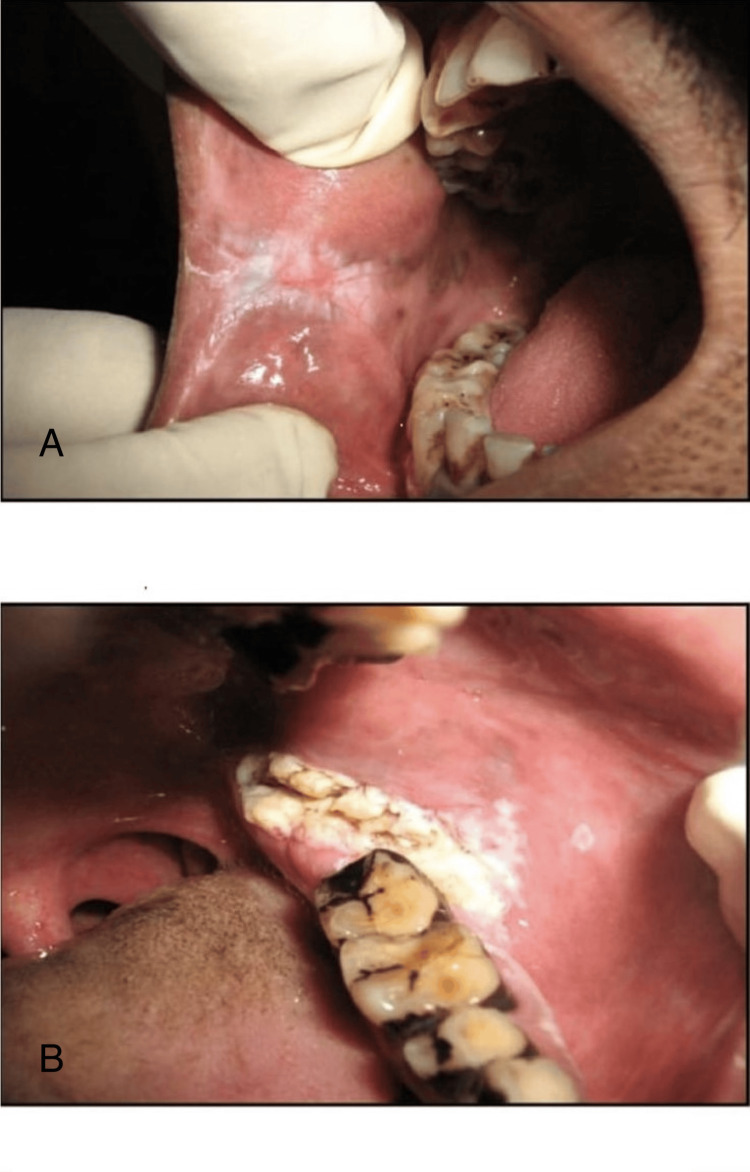
Intraoral photograph of oral leukoplakia and oral squamous cell carcinoma A: Intraoral photograph showing oral leukoplakia (OL) on the right buccal mucosa; B: Intraola photograph showing oral squamous cell carcinoma (OSCC) on the left retromolar area

**Figure 2:**
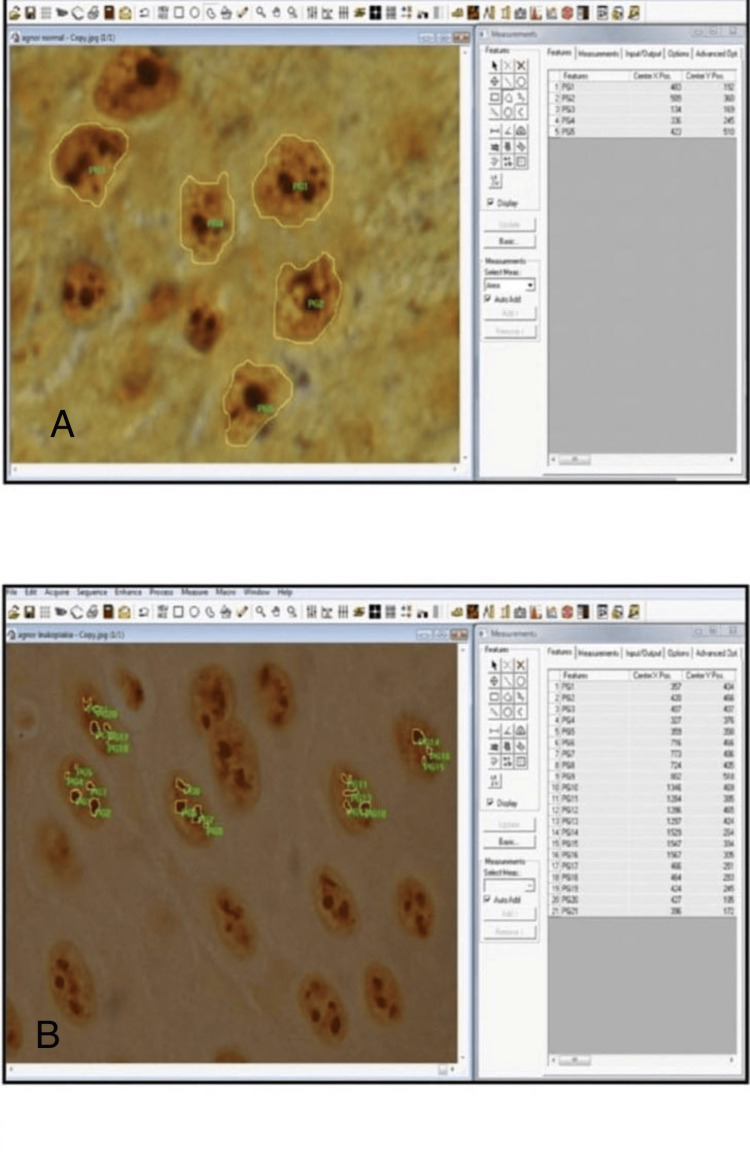
AgNOR stained section of normal oral mucosa and oral leukoplakia A: AgNOR stained normal oral mucosa (NOM) (measurement of nuclear area) (x100); B: AgNOR stained section of oral leukoplakia (OL) (measurement of single AgNOR area, total AgNOR area and number of AgNOR) (x100)

**Figure 3:**
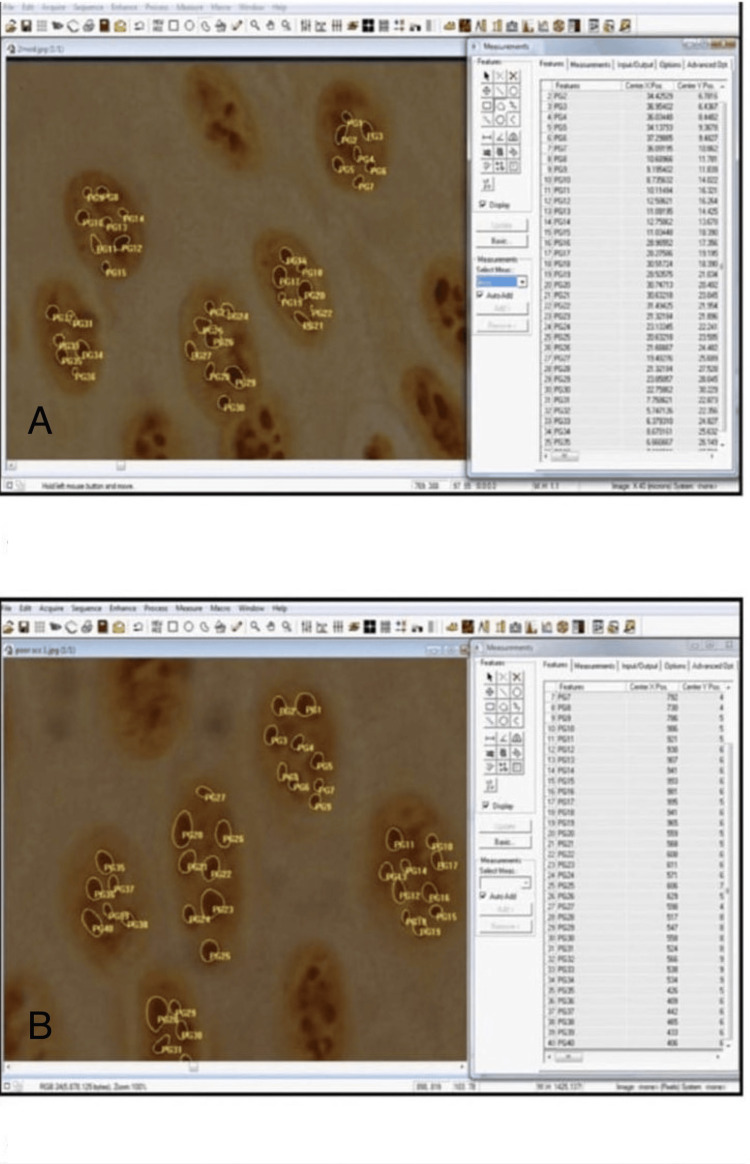
AgNOR stained section of moderately differentiated OSCC and poorly differentiated OSSC A: AgNOR stained section of moderately differentiated oral squamous cell carcinoma (OSCC) (measurement of single AgNOR area, total AgNOR area, and number of Agnor) (x100); B: AgNOR stained section of  poorly differentiated oral squamous cell carcinoma (OSSC) (measurement of single AgNOR area, total AgNOR area, and number of Agnor) (x100)

**Figure 4:**
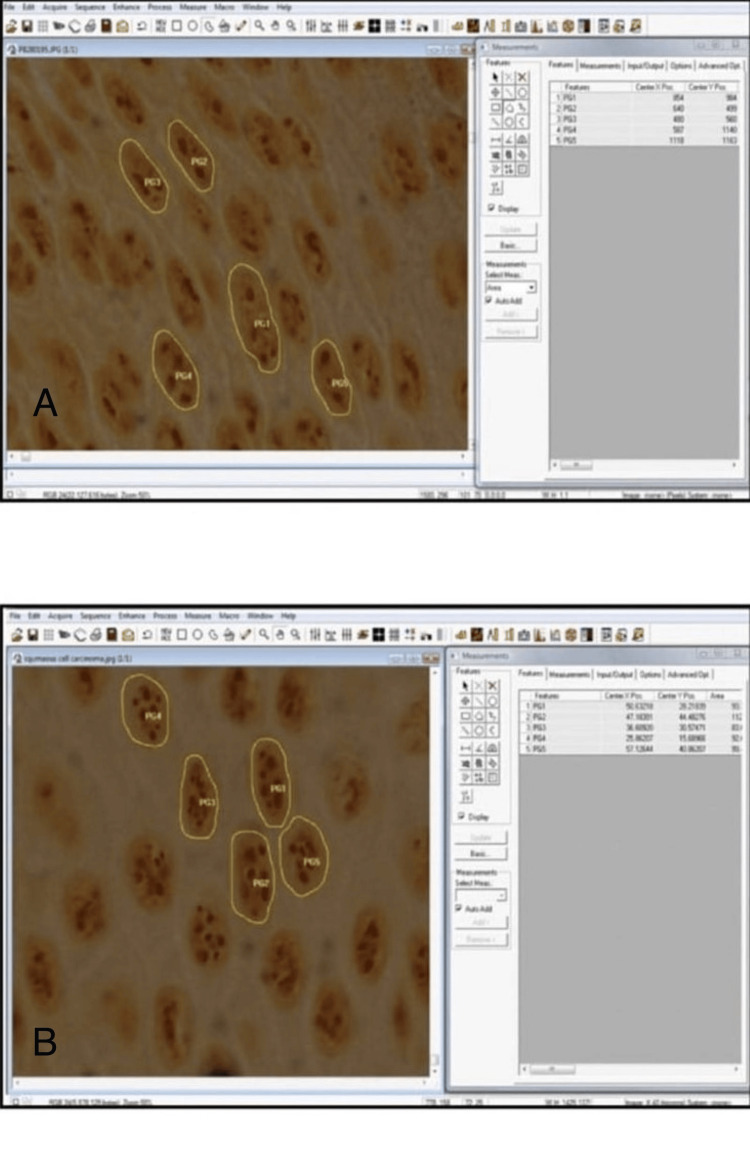
AgNOR stained section of oral leukoplakia and well differentiated OSSC A: AgNOR stained section of oral leukoplakia (OL) (measurement of nuclear area) (x100); B: AgNOR stained section of well-differentiated oral squamous cell carcinoma (OSSC) (measurement of nuclear area) (x100)

A total of 50 subjects distributed in three groups were analyzed including 20 OL, 20 OSCC, and 10 NOM for the control group. The AgNORs were evident as black "dots" in the brown-stained nucleus on a pale yellow background. Spherical-shaped or oval AgNOR dots were seen in normal oral mucosa (control) while in cases of leukoplakia (premalignant lesion), the AgNORs were not so round or oval but irregular in shape. In OSCC AgNORs were distributed throughout the nucleus, were in clusters, and were irregular in shape. The findings of the mean A Nuc, differences in mean A NOR, mean TA NOR, as well as the n NOR per nucleus, were compared in cases of OL, OSCC, and NOM (Figures 1, 2, 3, 4).

Table 1 depicts the findings of A Nuc, A NOR, TA NOR, and n NOR in cases of OL, OSCC, and control group. On statistical analysis, the association of the mean nuclear profile of OL and OSCC was found to be highly significant when compared to the control group (P value <0.01). F (ANOVA) for A Nuc, A NOR, TA NOR, n NOR was 9.836, 89.704, 52.533, 364.968 respectively.

**Table 1:** Mean nuclear profile of different groups

S.No.	Group	No. of subjects	A Nuc		A NOR		TA NOR		n NOR	
			Mean	SD (±)	Mean	SD (±)	Mean	SD (±)	Mean	SD (±)
1.	Control	10	36.19	7.83	3.45	0.37	5.27	0.67	2.58	0.13
2.	Study	40	52.16	21.73	2.19	0.68	9.06	2.94	6.82	1.30
	Oral leukoplakia	20	41.97	6.43	2.76	0.41	6.81	1.14	5.68	0.40
	Oral sq cell carcinoma	20	62.36	26.63	1.61	0.30	11.30	2.43	7.92	0.70
ANOVA (f-value)			9.836		98.704		52.533		364.968	
p-value			<0.001		<0.001		<0.001		<0.001	

Table 2 depicts the comparison of various parameters of morphometric analysis among cases of oral leukoplakia and oral squamous cell carcinoma with normal oral mucosa of controls. On comparing the findings of all four parameters i.e. A Nuc, A NOR, TA NOR, and n NOR were found to be statistically associated with the premalignant (oral leukoplakia) and malignant changes (oral squamous cell carcinoma).

**Table 2:** Comparison of normal oral mucosa with oral leukoplakia and oral squamous cell carcinoma (OSCC)

S.No.	Parameter	Control vs oral Leukoplakia		Control vs OSCC	
		"t"	"p"	"t"	"p"
1.	A Nuc	2.159	<0.05	3.019	<0.01
2.	A NOR	4.441	<0.001	14.525	<0.001
3.	TA NOR	3.920	<0.01	7.637	<0.001
4.	n NOR	23.665	<0.001	23.861	<0.001

## Discussion

It is well recognized that fundamentally all OSCCs are heralded by noticeable modifications in oral mucosa in the form of leukoplakia or erythroplakia etc. [10]. It is understood that documentation and supervising these potentially malignant disorders allow the clinician to detect and treat early stages of oral cancer [11]. Early detections of premalignant and/or neoplastic lesions are therefore essential for refining the long-term prospective of patients suffering from OSCC [12]. Certain cases of OL regress or stay inactive, but while many progress and are ultimately excised, 3-6% transform into OSCC [13]. It is, therefore, imperative to differentiate between these lesions as their management may differ accordingly [14].

Studies have suggested that AgNORs (replicatory markers) might be useful in the diagnosis of various neoplasms. OSCC can be distinguished from normal epithelium by AgNOR count. Some studies have indicated that AgNOR could distinguish mild and moderate epithelial dysplasia. The AgNOR technique, which is inexpensive and its results are easily reproducible, can be very effective in resource-poor settings [15].

Early detection of OSCC not only increases the survival rate but also improves the quality of life by reducing the need for aggressive and disfiguring treatments. Several reports have suggested that variations in AgNOR counts may reflect the state of activation or degree of malignancy of the lesion involved [16]. The AgNOR count is the most appropriate marker to differentiate between dysplastic and nondysplastic leukoplakia [17]. AgNOR analysis may be useful as a quantitative marker of incipient cellular alterations and hence would be helpful in assessing suspicious lesions and thus can be regarded as a valuable adjunct [18].

Many investigators have commented on the histopathology of OL and OSCC using mean AgNOR number and its significance. However, very few have undertaken studies using various parameters of AgNORs like A Nuc, A NOR, TA NOR, and n NOR.

In the present study, A Nuc of NOM cases showed a mean value of 36.19±7.83 mm2 which could be compared with other studies done by Cabrini et al. [19] who reported A Nuc of NOM to be 44.88±9.03 mm2, and Schwint et al. [20] who studied sections from such cases with a mean value of 44.88±9.03 mm2. In our study, the nuclear profile area from the section of OL cases showed a mean value of 41.97±6.43 mm2. This result could be compared to studies conducted by Ganesh et al. [21] who observed a mean value of 33.13±3.30 mm2. In the present study, the nuclear profile area for OSCC showed a mean value of 62.36±26.63 mm2 which can be compared with a study done by Cabrini et al. [19], who reported a mean value of 68.05±24.22 mm2. Hence, these above results showed a marked difference in mean nuclear area between NOM, OL, and OSCC thus, implying that the nuclear area increases as epithelial cells undergo malignant transformation.

In the present study, the single AgNOR profile area of NOM showed a mean value of 3.45±0.37 mm2. This was seen to be in accordance with the previous studies carried out by Cabrini et al. [19] and Chandak et al. [22]. Cabrini et al. [19] in their study showed that the single AgNOR profile area in NOM was 3.32±2.01 mm2 and Spolidorio et al. [19] showed that the mean value of the same was 3.17±0.57 mm2. The single AgNOR profile area of OL provided a mean value of 2.76±0.41 mm2 in the present study, which could be compared with the study conducted by Muzio et al. [23]. They showed that the mean value in leukoplakia with low, moderate, and severe dysplasia was 2.99±1.36 mm2, 5.01±1.04 mm2, and 4.03±0.85 mm2 respectively. Another study done by Chandak et al. [22] showed that the mean value of the single AgNOR profile area was 4.98±0.77 mm2. The single AgNOR profile area of OSCC showed a value of 1.61±0.30 mm2 in the present study. These results are compatible with similar studies undertaken by Schwint et al. [20] who reported a mean value of 1.65±0.61 mm2 in OSCC cases. 

In the present study, the TA NOR of NOM showed a mean value of 5.27±0.67 mm2 which could be compared with studies done by Schwint et al. [20]. who showed that the mean value of the same in NOM was 6.78±1.87 and Chandak et al. (2011) [22] reported a value of 4.28±0.97 mm2 in NOM cases. In the present study, the total AgNOR profile area (TA NOR) of OL showed a mean value of 6.81±1.14 µm2 as compared to studies carried out by Muzio et al. [20] and Chandak et al. [22]. In our study, the total AgNOR profile area of OSCC showed a mean value of 11.30±2.43 µm2 which could be compared with a study reported by Schwint et al. [20]. These results suggested that the total AgNOR profile area is increased in OL to OSCC when compared to NOM. The total AgNOR profile area showed an increased value from normal oral mucosa to oral squamous cell carcinoma because of an increase in the number of AgNORs per nucleus as the cells became atypical. This has also been discussed by various authors like Kanitakis et al. [24], Gilberto et al. [25], and Piffko et al. [26].

In the present study, the number of profiles of AgNOR per nucleus (n NOR) of NOM showed a mean value of 2.58±0.13. This was in concordance with a study reported by Cabrini et al. [19]. The mean value of the same in OL cases in our study was seen to be 5.68±0.40, which could be related to the study done by Chowdhry et al. [27] who observed a mean value of the same in epithelial dysplasia to be 5.61±4.63 and in candidal dysplasia to be 5.67±4.83. In our study, the number of profiles of AgNOR per nucleus (n NOR) of OSCC showed a value of 7.92±0.70. These results could be compared to other studies done by Cabrini et al. [19], Chowdhry et al. [27], and Schwint et al. [20].

Several studies have compared the mean count of n NOR in NOM, OL, and OSCC. These studies reported an increase in the mean count of n NOR from NOM, to OL and OSCC [19, 28-29].

Hence, the results in the present study suggested that the mean AgNOR count showed a higher value in OSCC when compared to NOM. This implied that the number of AgNORs in nuclei increases as epithelial cells undergo malignant transformation which indicated that mean AgNOR count may assist in ascertaining the prognosis of a dysplastic lesion.

## Conclusions

On comparison, statistical significance was revealed among different groups thus specifying that it is possible to identify and differentiate oral epithelial changes with the help of AgNOR morphometric analysis. The order of mean value of A Nuc, TA NOR, and n NOR in relation to the diagnostic groups were as follows:

Normal oral mucosa < Oral Leukoplakia (with dysplasia) < well-differentiated oral squamous cell carcinoma < Moderately differentiated oral squamous cell carcinoma < Poorly differentiated oral squamous cell carcinoma.

It suggested that OSCCs feature more irregular, smaller, and abundant AgNORs. This implies that as epithelial cells undergo dysplastic changes and then undergo malignant transformation, an increase in nuclear area is evident as represented morphometrically. AgNOR morphometric analysis may be useful to determine a cut-off value for identifying epithelial changes at the early stages of transformation, thereby helping to intervene with appropriate treatment before the disease progresses to overt malignancy.
